# Rapid Assessment Procedure for Loiasis and Mapping Lymphatic Filariasis: Two Perfect Illustrations of “To Be in English or Not to Be”

**DOI:** 10.1371/journal.pntd.0001863

**Published:** 2012-12-13

**Authors:** Bernard Carme

**Affiliations:** Laboratory of Parasitology and Mycology, Research Team EA 3593 and Centre d'Investigation Clinique - Epidémiologie Clinique des Antilles et de la Guyane (CIE 802 INSERM), Cayenne General Hospital and Faculty of Medicine, University Antilles Guyane, Cayenne, French Guiana; National Institute of Parasitic Diseases China CDC, China

## Abstract

Interest in filariasis has found a new impetus now that neglected tropical diseases have their own journal. However, some of the advances published in renowned international journals have completely ignored previous publications on the subject, particularly those in languages other than English. The rapid assessment procedure for loiasis and the mapping of lymphatic filariasis provide two perfect illustrations of this. This problem may seem a bit outdated, given that all “good authors” now publish exclusively in English. It certainly is outdated for most areas of medicine. But, surely, this should not be the case for neglected tropical diseases, for which certain long-standing findings are every bit as important as what may be presented as new discoveries. One possibility would be for certain journals, such as *PLOS Neglected Tropical Diseases*, to include a specific heading permitting the publication in English of older studies that initially appeared in a language other than English. The texts would be English versions respecting the entirety of the original text. Submission should be accompanied by a presentation of the problem, with details and explanatory comments, with submission at the initiative of the authors of the former article in question or their students or sympathizers.

Interest in filariasis has found a new impetus now that neglected tropical diseases (NTDs) have their own journal. However, some of the advances published in renowned international journals have completely ignored previous publications on the subject, particularly those in languages other than English. This Viewpoint article is intended to make us ponder the issue of a language gap or discrimination existing in publishing outcomes and reference citations. This is also the question of deleterious effects of the obligation “to be in English or not to be”.

The rapid assessment procedure for loiasis (RAPLOA) and the geographical distribution of lymphatic filariasis provide two perfect illustrations of this.

The RAPLOA has recently been widely used to determine the regional endemicity of loiasis and to update existing endemicity data for this disease over its global distribution range. This important work has been recently published in *PLOS NTDs* [1]. The determination, within a population of the prevalence or, preferably, the annual incidence of episodes, of conjunctival migration by adult worms is a simple, non-invasive, relatively sensitive and specific method for evaluating the endemicity of *Loa loa*. This approach has proved particularly useful in areas in which both loiasis and onchocerciasis are observed: the mass treatment program to control onchocerciasis is based on the use of ivermectin and there is a risk of adverse treatment outcomes in patients carrying large numbers of *L. loa* worms [2]. In regions of high endemicity, the correlation between the conjunctival migration index and the microfilarial index is strong overall, both for villages and for age groups. Its use as an epidemiological index was clearly proposed in a publication in 1994 [3]. However, as this article was published in French, in *Médecine Tropicale (Marseille)*, it has never been cited, despite being listed in international databases, including PubMed. A poster communication concerning the same issue had no real impact either, despite being presented at an international congress [4]. The origin of this new epidemiological index (RAPLOA) is systematically attributed to two World Health Organization (WHO) publications in 2001 [5] and 2002 [6]. It is true that the studies reported in these publications validated the concept at a large scale and in different endemic foci.

The usefulness of specific clinical manifestations (eye worm and Calabar swelling) to assess *L. loa* had been recommended by different authors as early as 1950 [7]. But the correlation between the microfilarial index and the frequency of ocular migration has not been studied, and even less attention was paid to the notion of an epidemiological index until the epidemiological studies carried out in Congo Republic (former People's Republic of the Congo) during the 1980s [8]. However, the index as such was clearly defined in 1994 [3]. Here is a direct translation of an excerpt of the French text published in 1994: “For loiasis, the usual parasitological indices (microfilarial index and mean microfilarial density) are the only measures recognized as providing information about the level of endemicity in humans. In addition to requiring blood samples standardized in terms of both volume and sampling time, these indices do not reflect the real level of parasitism, given the high frequency of infected subjects without microfilaria in the blood. Subjects infested with mature, fertile adult worms, as demonstrated by the removal of a subconjunctival female containing microfilaria from a patient with no detectable microfilaria in the blood, are frequently observed. Two symptoms are both specific and frequent in infected subjects both with and without microfilaria in the blood: subconjunctival migration of an adult worm and elusive, migrating edemas of the hands, wrists and lower part of the forearm” [9]. “The index of subconjunctival filarial migration over the preceding year is particularly useful, because it correlates well with the microfilarial index but is more sensitive” (Figure 1). “Its determination involves precise questioning of the patient, which can be facilitated by the use of a demonstration chart, with diagrams and photographs” (see Figure 2).

**Figure 1:**
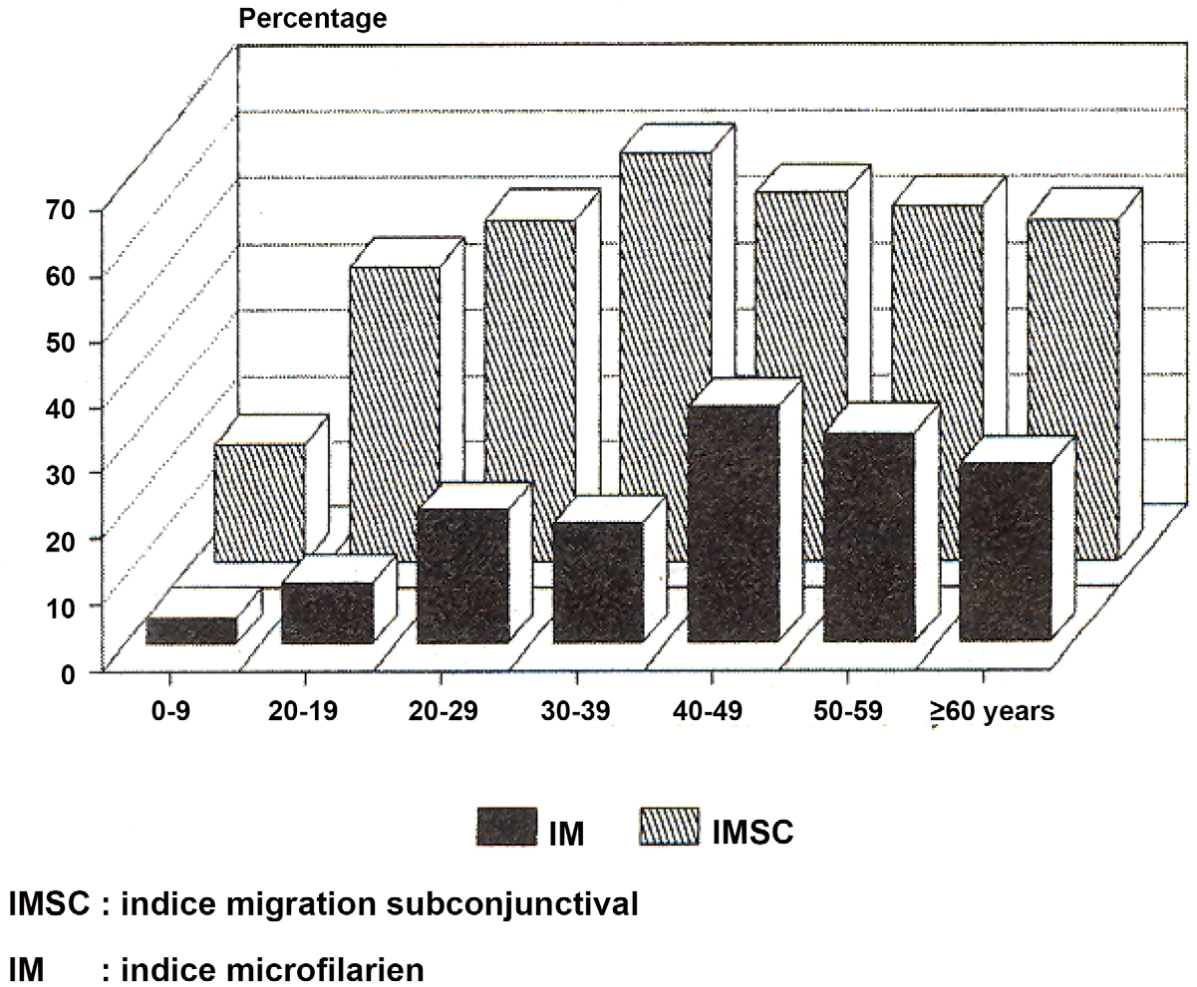
Index of the subconjunctival migration of *Loa loa* adult worms and microfilarial index. Reproduced from *Medicine Tropicale* [3], released under CC BY 2.0 by *Medicine Tropicale*. IMSC = Indice de Migration Sous-Conjonctivale in French and Index of the SubConjunctival Migration in English. IM = Indice Microfilarien in French and Microfilarial Index in English.

**Figure 2:**
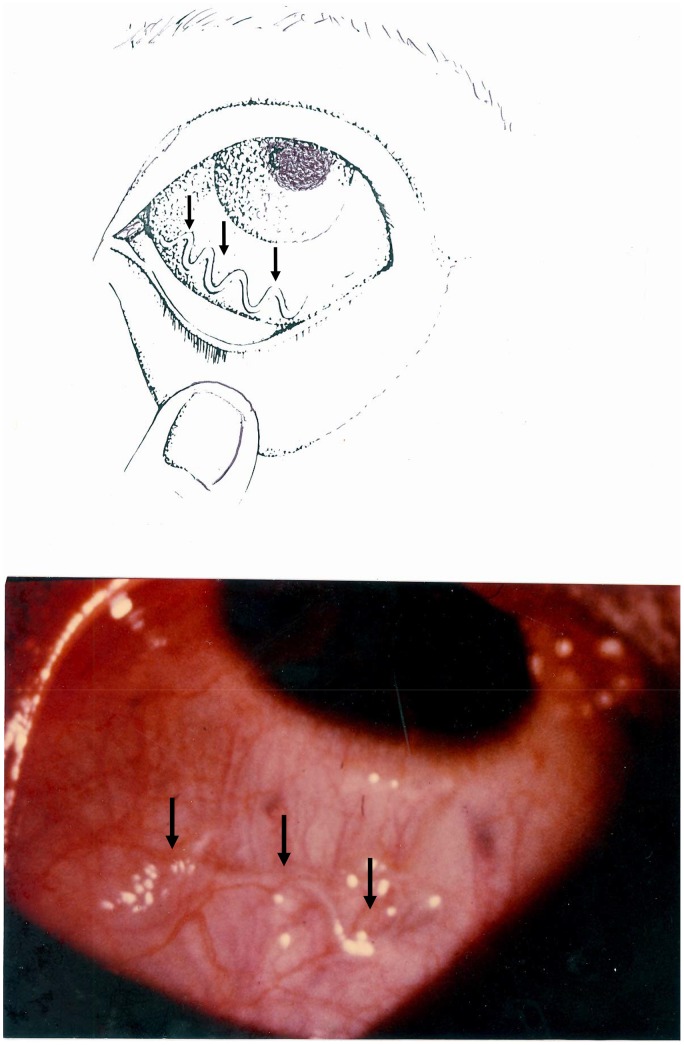
Illustration of the passage of an adult worm (*Loa loa*) across the eye. This illustration (diagram and photograph) was made for presentation to patients questioned in endemic regions.

The conclusion of this article was formulated as follows: “Screening for foci of filarial endemicity could be improved by the use of a simplified method and the validation of simple, inexpensive indices. Once these foci have been identified, a more precise evaluation can be carried out.”

What is most astounding about the two WHO publications cited as the origin of this “new epidemiological index” [5], [6] is that the principal authors come from French-speaking African countries and/or work in or with this institution. They would therefore have been able to understand articles written in French. Furthermore, the WHO has a long-standing culture of multilingualism, particularly in English and French.

Against this background, the rejection by the *Bulletin of the World Health Organization* and by other international journals published in English of an opinion article dealing with this issue and using the example of lymphatic filariasis does not seem to be justified, and is another illustration of “to be in English or not to be.”

Indeed, filariasis due to *Wuchereria bancrofti* is systematically described as endemic in Congo and Gabon, two French-speaking countries, in non-specialist works on tropical medicine and in more specialist publications (WHO) despite a total absence of epidemiologic studies and/or confirmed case report over the last 30 years to prove it. What is certain is that no case was found when the last studies were conducted in these countries at the end of the 1970s and during the 1980s but unfortunately published in French. The studies that we carried out in the Congo as part of the National Project on Onchocerciasis and Other Filariases (between 1982 and 1987) confirmed the presence of four types of human filariasis: onchocerciasis, loaiasis, and the filariases caused by *Mansonella perstans* and *M. streptocerca*. There was a total absence of confirmed cases of lymphatic filariasis (bancroftosis). In this case, it is not a question of the attribution of merit for a particular “discovery”, but of basic knowledge of the geographic distribution of a scarcely studied disease, lymphatic filiariasis, in French Central Africa. Taking into account only publications in English, even older and poorly structured data, have been, in our opinion, a source of confusion and has led to false conclusions being drawn about the distribution range of this disease. This undoubtedly highlights the need to update knowledge by carrying out prospective studies (which seem to be underway), but these studies do not seem to be considered a matter of priority given the low levels of resources available and current health priorities.

This has drawn us to publish this article in a French-language journal, but together with an entire translation into English [10]. Despite the bilingual nature of this publication, the international PubMed database identifies this article as being published in French, effectively ensuring that it will never be consulted, a classic “catch 22” situation! Indeed, this reference has never yet been cited by another author in a journal published in English. It may be that publication of an article in another language than English makes it more likely that it will not be cited, even if the authors of a subsequent article have access to the journal in which it was published and can understand the language used. Here, we begin to encroach on ethical problems and it is probably best not to delve too deeply. However, suffice it to say that the limited dissemination of publications in a language other than English may account for such equivocal attitudes.

The problem is not a rivalry between French and English, but the confrontation between English and all other languages of the world. Moreover, the problem is undoubtedly worse for works published in non–Western European languages such as Chinese, Russian, and Japanese, which are arguably even less accessible.

All things considered, this problem may seem a bit outdated, given that all “good authors” now publish exclusively in English. It certainly is outdated for most areas of medicine, where everything that is old is assigned to being nothing more than the history of medicine. But, surely, this should not be the case for NTDs, for which certain long-standing findings are every bit as important as what may be presented as new discoveries.

One possibility would be for certain journals, such as *PLOS NTDs*, to include a specific heading permitting the publication of older studies that initially appeared in a language other than English (and are therefore currently little known). The texts included in this heading would essentially be English versions of these articles previously published in other languages, respecting the entirety of the original text.

This would concern studies considered of importance because they highlight a point that remains unclear or describe an aspect considered innovative in a review but for which the originality of the article is due more to an incomplete reference list than to a true advance in knowledge. These articles should be judged in light of the knowledge and technical and methodological means available at the time at which they were initially published. Submission should be accompanied by a presentation of the problem, with details and explanatory comments, with submission at the initiative of the authors of the article in question or their students or sympathizers.
